# Survival of the enveloped bacteriophage Phi6 (a surrogate for SARS-CoV-2) in evaporated saliva microdroplets deposited on glass surfaces

**DOI:** 10.1038/s41598-020-79625-z

**Published:** 2020-12-29

**Authors:** Aliza Fedorenko, Maor Grinberg, Tomer Orevi, Nadav Kashtan

**Affiliations:** Department of Plant Pathology and Microbiology, Robert H. Smith Faculty of Agriculture, Food, and Environment, Hebrew University of Jerusalem, 76100 Rehovot, Israel

**Keywords:** Bacteriophages, Environmental microbiology, Virology, Microbiology, Infectious diseases

## Abstract

Survival of respiratory viral pathogens in expelled saliva microdroplets is central to their transmission, yet the factors that determine survival in such microdroplets are not well understood. Here we combine microscopy imaging with virus viability assays to study survival of three bacteriophages suggested as good models for respiratory pathogens: the enveloped Phi6 (a surrogate for SARS-CoV-2), and the non-enveloped PhiX174 and MS2. We measured virus viability in human saliva microdroplets, SM buffer, and water following deposition on glass surfaces at various relative humidities (RH). Saliva and water microdroplets dried out rapidly, within minutes, at all tested RH levels (23%, 43%, 57%, and 78%), while SM microdroplets remained hydrated at RH ≥ 57%. Generally, the survival of all three viruses in dry saliva microdroplets was significantly greater than those in SM buffer and water under all RH (except PhiX174 in water under 57% RH survived the best among 3 media). Thus, atmosphere RH and microdroplet hydration state are not sufficient to explain virus survival, indicating that the virus-suspended medium, and association with saliva components in particular, likely play a role in virus survival. Uncovering the exact properties and components that make saliva a favorable environment for the survival of viruses, in particular enveloped ones like Phi6, is thus of great importance for reducing transmission of viral respiratory pathogens including SARS-CoV-2.

## Introduction

Microdroplets expelled from the human respiratory tract into the air through coughing, talking, and breathing are considered a key source of transmission of respiratory viruses including SARS-CoV-2^1,2^. These microdroplets, ranging in size from a few micrometers up to millimeters^3–5^ travel in the air, and some of them—larger ones in particular—settle on surfaces^2^. Thus, inanimate surfaces are a potential route of virus transmission^1,2,6,7^. The factors that affect virus survival in droplets that settle on surfaces are complex and not well understood. Survival is believed to depend upon the physicochemical characteristics and hydration conditions of the immediate microscopic environment of the virion. These in turn are determined by several factors, including the composition of the fluid comprising the droplets, the surface properties, the ambient temperature, and the relative humidity (RH). A preeminent source for expelled droplets is human saliva, a complex solution that contains salts, a variety of proteins, and surfactants^8–11^. It has been suggested that micrometer-sized dry deposits of saliva droplets on surfaces protect virions^12^. Still, it is not clear how well viruses survive in saliva microdroplets following their deposition on surfaces, and what factors are at play therein. The impact of RH on the stability and viability of SARS-CoV-2 and that of other enveloped viruses has been studied mostly in airborne droplets or aerosols^13–19^ and less on surfaces^20–22^.

While survival varies between virus species, increased survival at both low (< 40%) and very high (> 90%) RH is often observed, with decreased survival at intermediate RH levels^11,13,14,17,20,23^. The underlying mechanism of this U-shaped survival as a function of RH is not clear^16^. Only a few studies have attempted to gain mechanistic understanding of how RH affects virus stability in microdroplets. Prominent among these are the pioneering studies of the Marr group^5,16,18,24^, which point to the role of the solvent composition, evaporation dynamics, and RH on virus survival in aerosols and sessile droplets.

A key factor determining the evaporation rate and equilibrium hydration level of drying droplets on surfaces (and in air as well) is the deliquescence property of solutes, or mostly highly hygroscopic salts^25–27^. Accordingly, although many surfaces in the indoor environment appear dry, they are in fact covered by thin liquid films and micrometer-sized droplets, invisible to the naked eye, known as *microscopic surface wetness* (MSW) ^28^. MSW can be considered the “envelope” that accommodates microorganisms on surfaces, and as such has profound impact on many aspects of microbial life. For example, it can protect microbes from complete desiccation. However, MSW is a harsh micro-environment that differs in its properties from those of bulk liquid. MSW inherently arises from drying liquids that evaporate on a surface. This drying process is accompanied by physicochemical changes such as solute concentrations, pH, reactive oxygenic species, surface tension, and others. At the microscale, gradients, local densities, and surface irregularity introduce heterogeneity to MSW environments^11,29–31^. Collectively, MSW imposes severe stresses on microbes therein—including viruses—and affects their survival^11,18,20,32,33^.

The current study was motivated by the urgent call for the scientific community to address SARS-CoV-2 spread. Like many other viruses, SARS-CoV-2 has been shown to survive well and remain viable on various surfaces, e.g., metal, glass, and plastic, for up to several days^19,34–39^. While not necessarily viable, SARS-CoV-2 RNA has been detected on surfaces in contaminated sites such as hospitals^40–43^. Here, we aim to explore the link between microscopic surface wetness and virus survival therein. We focus on two variables that directly affect MSW—solution composition and RH levels—and seek to determine whether and how it is reflected by virus survival trends.

We use three well-studied bacteriophages previously suggested as good, safe, and easy to work with, as model surrogates for studying surface and air survival of pathogenic viruses^44–46^: the enveloped Phi6, and two other tailless non-enveloped model bacteriophages as a reference. Phi6 is a dsRNA phage of the *Cystoviridae* family that has been suggested as a good surrogate for studying enveloped RNA viruses including SARS coronaviruses^11,14,44,47–49^; similar to SARS-CoV-2, it is enveloped by a lipid membrane, has spike proteins, and is of similar size (~ 80–100 nm). The other virus strains that we used are the well-studied, non-enveloped MS2 (ssRNA; *Leviviridae*)^45,50–52^ and PhiX174 (ssDNA; *Microviridae*)^45,46,53^. To better understand how RH and the solution composition of microdroplets affect virus survival on surfaces, we combine microscopy imaging to assess MSW state with plaque assays to determine virus survival. We compare survival in ‘sprayed’ microdroplets of three suspensions: human saliva, SM buffer, and ‘pure’ water under a range of RH (23–78%) relevant to most indoor environments. The link between the microscopic environment of viruses, including hydration conditions, and virus viability, is discussed.

## Results

**Figure 1:**
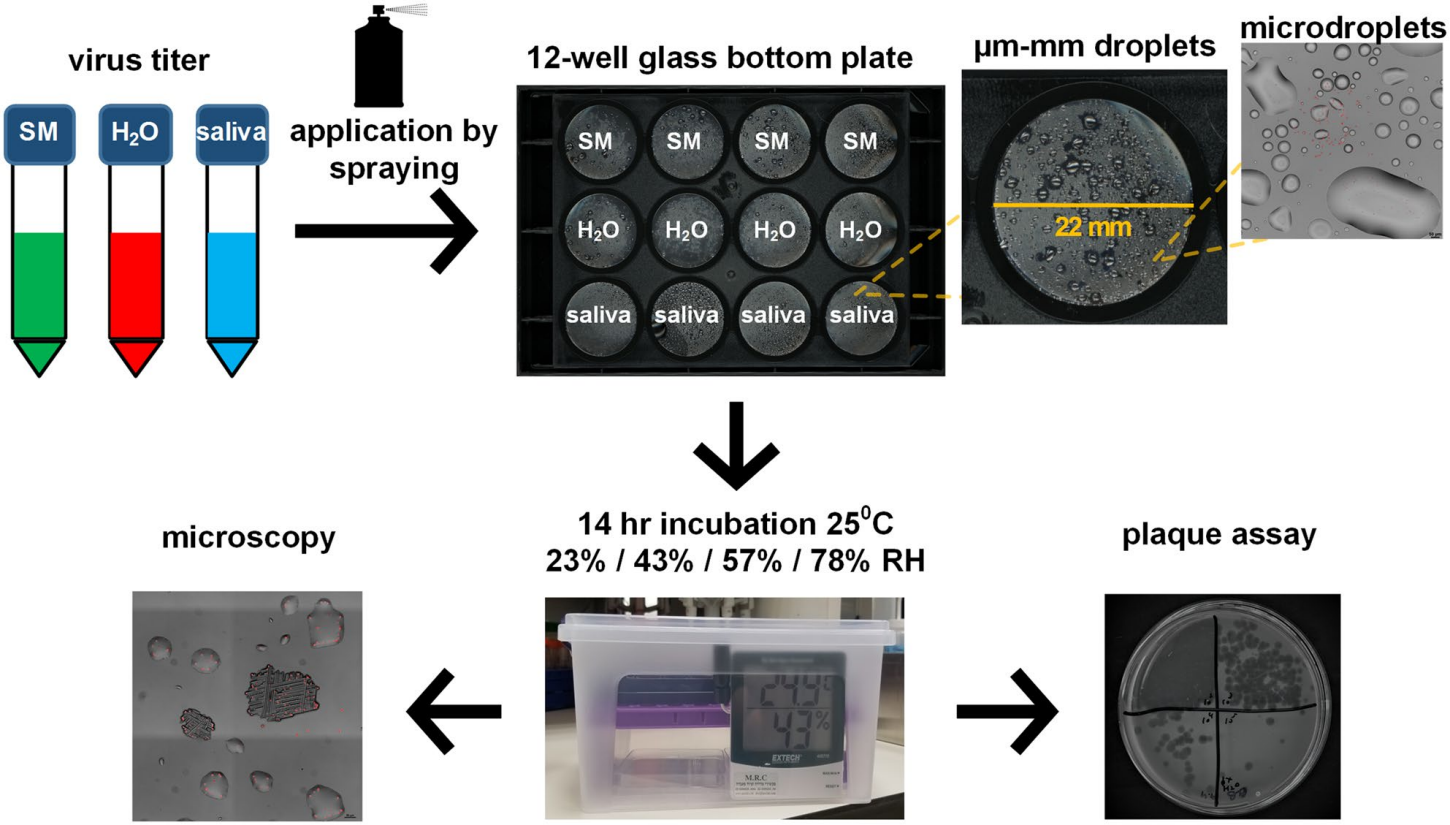
Schematic illustration of the experimental workflow. Phage suspended in SM buffer, H_2_O, and human saliva were applied onto a glass-bottom 12-well plate by a spraying device, resulting in microdroplets ranging in size from ~ 10 microns to ~ 1 mm in diameter. Plates were transferred to sealed containers that maintained specific RH conditions (using saturated salt solutions), and the containers were placed in an incubator to maintain constant temperature (25 °C) for 14 h. At the end of the incubation period, the surface of the plates was imaged by microscopy, and subsequently the wells were re-suspended and phage concentration was determined by the plaque assay.

**Figure 2:**
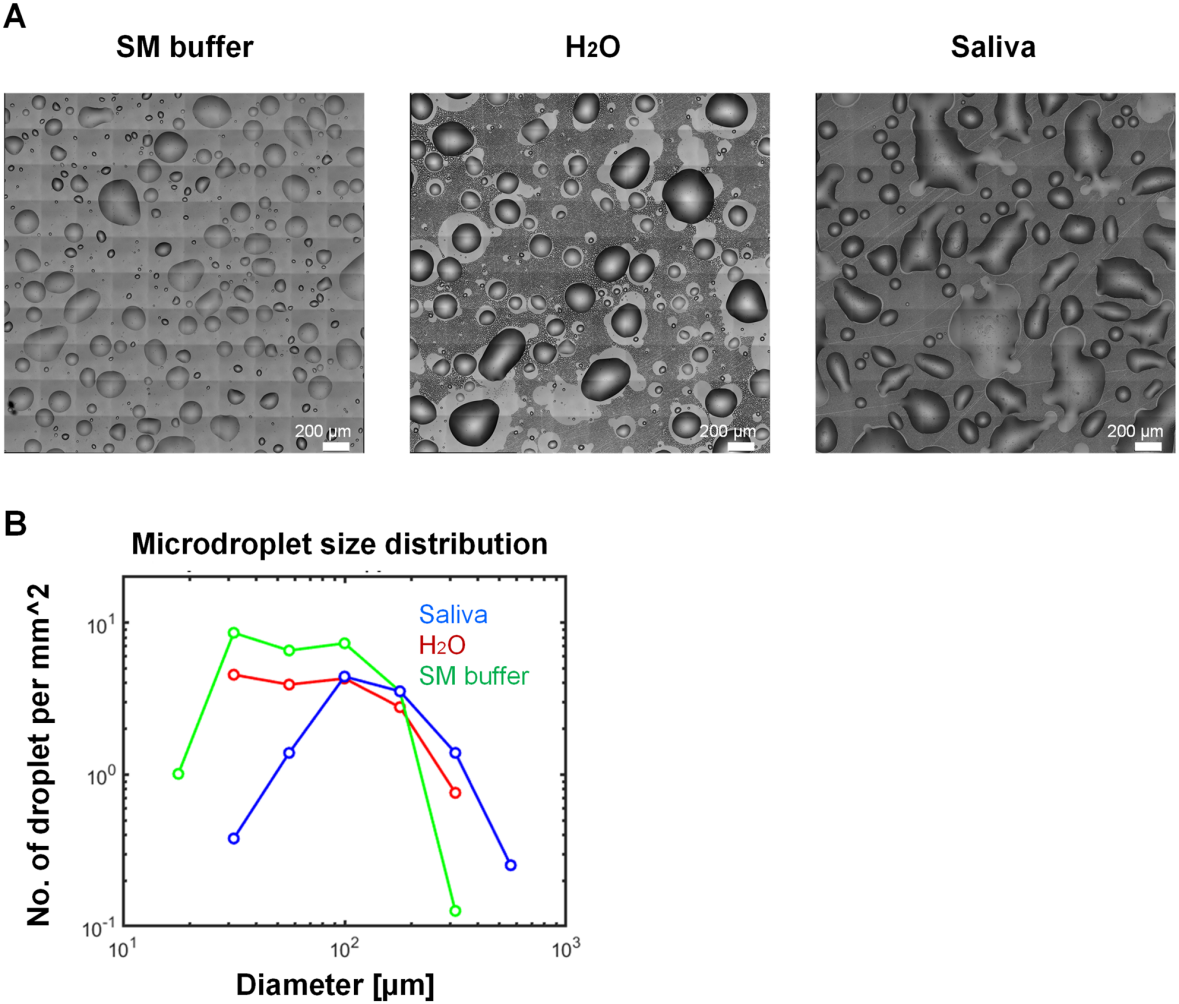
Representative microscopy images of sprayed SM, H_2_O, and human saliva on a glass surface. (**A**) typical shape and size of SM buffer, H_2_O, and saliva immediately after spray application onto a glass surface. (**B**) Droplet size distribution calculated based on the images shown in Panel A. Typical droplet size is between a few tens of microns to hundreds of microns in diameter, which is within the range of expelled saliva microdroplets that are expected to gravitate more rapidly toward surfaces (as opposed to aerosols, which remain suspended in the air for longer).

To study virus survival in microdroplets deposited on a smooth, inanimate surface, we sprayed Phi6, MS2, and PhiX174 viruses suspended in three aqueous media—human saliva, water, and SM buffer—on glass-bottom 12-well plates (Fig. 1, “Methods”). Fluorescent beads (2 µm in diameter) in an equivalent concentration to those of the Phi6 and MS2 viruses (~ 10^6^/mL), were added to the suspension. Sprayed microdroplet size ranged between tens to hundreds of microns in diameter (Fig. 2A,B), which falls within the range of respiratory fluid microdroplets exhaled while coughing, speaking, and breathing^4,5^ and gravitating toward surfaces (i.e., not the < 5 µm aerosols). The sprayed well plates were placed under constant temperature and RH conditions (23%, 43%, 57%, 78%) for 14 h, and subsequently microscopy images were taken and virus survival was estimated by the plaque assay using the corresponding bacterial host as a reporter for virus viability (Fig. 1, “Methods”).

**Figure 3:**
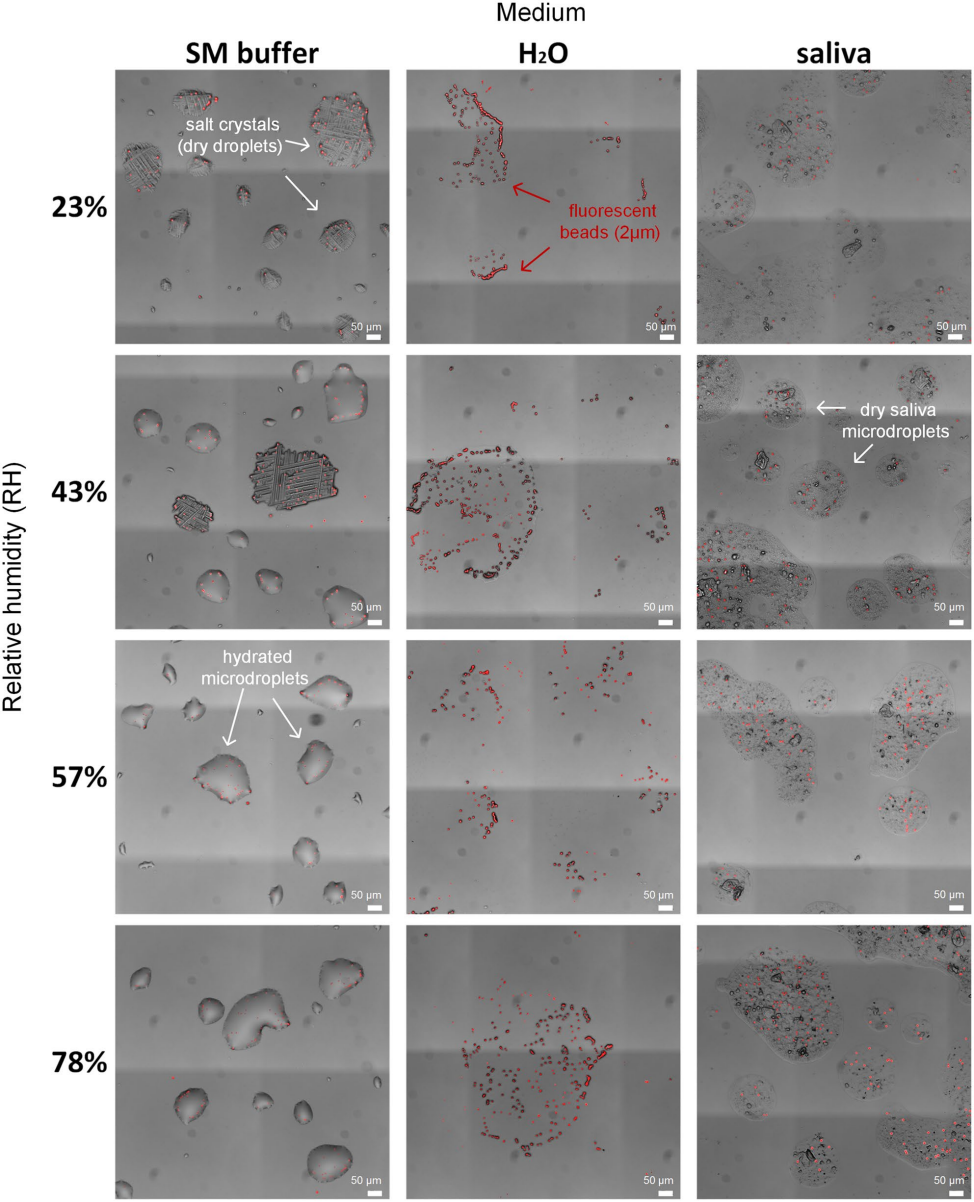
Microscopy images of microdroplets deposited on the glass-bottom well plates after 14 h incubation at a range of RH levels. SM buffer droplets remained hydrated at 57% RH and above, partially hydrated at 43% RH, and completely dry at 23% RH (medium salt crystallization can be seen in dried droplets). H_2_O and saliva droplets appeared to be dry under all tested RH conditions. Fluorescent micro-beads (2 µm) (red) were added at a concentration similar to the virus concentration, and thus may help to visualize the expected distribution of viruses within evaporated microdroplets.

To better understand the microscale hydration state that viruses experience, we first examined the surfaces under the microscope 14 h post deposition. Representative images of the surface at t = 14 h are shown in Fig. 3. The saliva microdroplets dried out rapidly, within minutes (see Supp. Video 1), and appeared completely dry at all tested RHs. As can be seen in Fig. 3, saliva microdroplets left a thin layer deposit (~ 3–5 µm thickness) of dry matter with aggregated substances (some of these aggregates are salt crystals, but some of them are not, as observed by Vejerano et al. ^11^). Beads were dispersed fairly uniformly within these microdroplet deposits, as previously demonstrated for virions^11^. While the beads are at least an order of magnitude larger than individual virions, their visualization can help us grasp the concentration of virions in droplets, and possibly their spatial distribution (assuming lack of virion aggregation—see “Discussion”). We estimated ~ 10 virions in a 100-µm droplet (see “Methods”), which is consistent with the observed bead distribution (Fig. 3).

Water microdroplets dried out rapidly at all RH levels (Supp. Video 2), as expected, as the water suspension contained very low concentrations of deliquescent solutes (e.g., salts). At RH = 78%, some tiny microdroplets (~ few µms in diameter) were observed around single beads or small bead clusters (Fig. 3). These tiny water microdroplets were likely retained due to strong capillary forces. In contrast, SM buffer microdroplets were hydrated at 57% RH and above (Fig. 3). Stable microdroplets of tens of µms in diameter could be clearly seen (Fig. 3). At RH = 43%, some of the microdroplets, but not all, dried out, and salt crystals were observed. At RH = 23%, all microdroplets dried out, and only salt crystals were seen (Fig. 3). These observations can be explained by the efflorescence point of NaCl, the major solute in SM buffer: ~ 45–48% RH ^54^.

**Figure 4:**
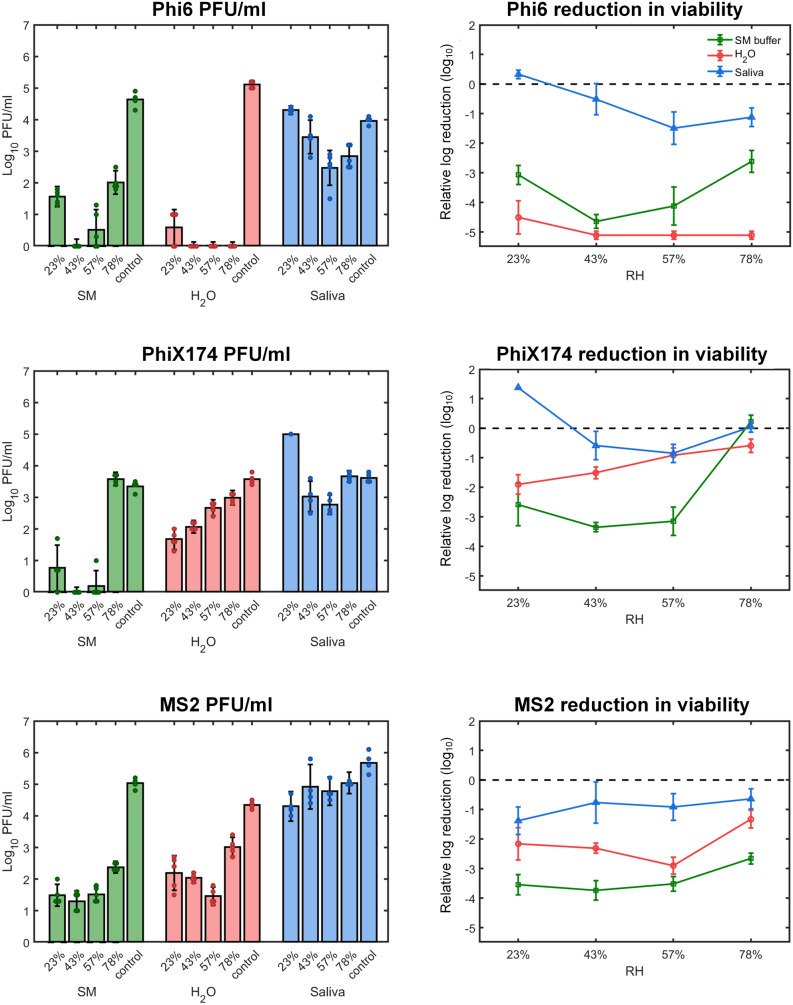
Survival of Phi6, PhiX174, and MS2 respectively as a function of RH and medium type on glass surface. Left panel: Bacteriophage titer concentration (PFU/mL) after 14 h incubation at 23%, 43%, 57%, and 78% RH at 25 °C. A 10^5^–10^6^ titer of bacteriophage suspended in SM buffer, H_2_O, or human saliva was applied by spraying on a glass surface. A control sample was left suspended in the spraying device for the duration of the incubation period. Following the incubation period, phage concentration was determined by plaque assay (mean ± SD of four replicates; points represent individual replicates). Right panel: Relative log reduction of viability of Phi6, PhiX174, and MS2 in SM buffer (green), H_2_O (red) and saliva (blue) (mean ± SD of four replicates) in evaporated microdroplets. Each data point is derived by subtracting the log value of the inoculum from the log value of the virus titer at each RH.

Next, we estimated virus survival under all tested conditions. The content of each well was suspended, and viral viability in the suspensions were evaluated (PFU/mL) by the plaque assay (Fig. 4 and “Methods”). Virus viability in sprayed droplets (saliva, water, and SM buffer) was compared with a control made from the same preparation that was kept in sealed tubes throughout the duration of the experiment, and then sprayed after 14 h and immediately re-suspended. Strikingly, the highest survival of all tested viruses was found in evaporated saliva microdroplet deposits, compared to the other media at any given RH levels. The exception was PhiX174, which had similar levels of survival in saliva and water at 57% RH and saliva and SM buffer at 78% RH respectively. Survival in saliva of all three viruses was relatively high, with less than 1.5 log reduction in viability, compared to the control. Phi6 survival ranged between a minimum of ≈ 3 × 10^2^ PFU/mL (RH = 57%) to a maximum of ≈ 2 × 10^4^ PFU/mL (RH = 23%) after 14 h in evaporated saliva droplets, compared to ≈ 1 × 10^4^ PFU/mL in the control (Fig. 4). The higher survival at RH = 23% might be explained by lower loss in the resuspension after spraying, compared to control. Similar to Phi6, PhiX174 survival in saliva droplets was lowest at RH = 57% with ≈ 6 × 10^2^ PFU/mL and highest at RH = 23% (> 1 × 10^4^ PFU/mL), again with higher survival than in the suspended medium (4 × 10^3^ PFU/mL). MS2 survival in saliva microdroplets increased with RH, between ≈ 2 × 10^4^ PFU/mL at RH = 23% to ≈ 10^5^ PFU/mL at RH = 78%, compared to 5 × 10^5^ PFU/mL in bulk (Fig. 4). The correlation between reduction in viability in saliva (in log10 values) varied between the phages (Supp. Figs. S1, S2). While Phi6 had negative correlation with weak regression correlation (with R^2^ = 0.52), MS2 showed positive correlation with weak linear correlation (R^2^ = 0.31). PhiX174 showed a non-monotonous U-shaped survival as a function of RH, with close to zero correlation and low R^2^ values (R^2^ = 0.008).

The fact that the water microdroplets were dry at all RH levels, appears to have a large impact on the enveloped virus Phi6, which showed more than 4 log reduction in viability (Fig. 4), with zero observed plaques at RH ≥ 43%. In contrast, the two non-enveloped viruses exhibited lower log reduction in viability of about 1–3 (> few hundreds PFU/mL compared to ≈10^4^ PFU/mL in the control, Fig. 4). While PhiX174 survival in evaporated water droplets was linearly correlated with RH (R^2^ = 0.87, Supp. Figs. S1, S2), hydration conditions did not show any noticeable change (Fig. 3). Overall, PhiX174 showed significantly lower viability in evaporated water microdroplets in comparison to saliva microdroplets, except comparable survival at 57% RH (Fig. 4, Supp. Fig. S3). MS2 survival in evaporated water microdroplets was significantly lower than that in saliva (Supp. Fig. S3), with a U-shaped RH dependence (Fig. 4, Supp. Fig. S1).

In the SM buffer, while microdroplets remained hydrated at 57% and 78% RH (Fig. 3), survival of all three viruses was significantly lower than in saliva under the corresponding RH levels (t-test, P < 0.05, See “Methods”, Supp. Fig. S3). The only exception was PhiX174, which exhibited similar survival levels in SM and saliva at 78% RH. Overall, Phi6, PhiX174, and MS2 in SM buffer deposits showed 3–4 log reduction in viability (reflected by no more than tens to hundreds of PFU/mL) across the tested RH range, exhibiting the familiar U-shaped trend (showing R^2^ < 0.5, Supp. Fig. S1).

## Discussion

Aiming to obtain some mechanistic insights into factors that determine virus survival in microdroplets deposited on surfaces, we explored a wide range of RH, three types of solutions, and three model viruses (enveloped and non-enveloped). Combining surface microscopy imaging and the virus viability assays, we were able to explore the link between the tested variables as manifested with respect to microscopic hydration state and virus survival. In summary, we observed significantly higher survival of all three viruses in evaporated saliva microdroplets in comparison to SM buffer and water, 14 h after deposition on a glass surface. The only exceptions were for Phix174 at the higher range of tested RH levels, where survival in saliva was comparable to that of other media. Phage survival in saliva microdroplets as a function of RH showed different trends (at the studied range of RH levels): Phi6 survival decreased with RH, MS2 survival increased with RH, and PhiX174 showed a U-shaped survival-RH relation.

Our results indicate that RH and hydration conditions are not sufficient to explain virus survival in microdroplets deposited on surfaces. For example, the log reduction of Phi6 at 78% RH was ≈ 1, ≈ 3, and ≈ 5 when suspended in saliva, SM buffer, and H_2_O respectively (Fig. 4). This implies that the physicochemical changes that characterize a microdroplet’s drying process have pivotal implications for virus survival. Thus, the effect of RH on virus survival is context dependent. Likewise, SM droplets were hydrated at 78% RH and completely desiccated at 23% RH. Nonetheless, Phi6 survival in SM droplets under these widely differing hydration states was comparable. We conclude that water availability and hydration status of a droplet cannot explain virus survival on its own. The observation that at a given RH, the microscopic hydration conditions of deposited droplets of various media can differ so widely (see along the rows of Fig. 3) suggests that RH does not directly affect virus stability and viability in drying microdroplets deposited on surfaces, but rather RH indirectly affects survival through its effect on physicochemical conditions at the scale that matters for viruses (~ µm).

Remarkably, both enveloped and non-enveloped viruses survive well in evaporated human saliva microdroplets deposited on glass surfaces at a wide range of RH levels. The observation that saliva droplets were completely dry even at 78% RH was somewhat surprising, as we expected saliva to effloresce at 40–50% RH ^54^. Human saliva is a complex fluid that somehow protects viruses in drying droplets and aerosols ^11,12^. A rough approximation estimates that in our experiments, droplets of ~ 100 microns in diameter contained around 10 virions (see “Methods”). Thus, the mass of salts, proteins, and surfactants in these droplets are a few orders of magnitude higher than is the total virion mass^11^. As suggested by Marr et al., association between virion and saliva proteins (or other components) can protect the virions for prolonged periods (even days) in such dried saliva droplets on surfaces. In addition, the lower content of inorganic salts in saliva^55^ than that in SM buffer may explain part of the large differences in virion survival between the two solutions.

A second remarkable result is the very low survival (> 4 log reduction) of Phi6 in the evaporated water microdroplets, regardless of RH. This result may indicate damage associated with the lipid membrane of the enveloped phage under these conditions. We note that in the control bulk water solution, survival of Phi6 was high and comparable to that in SM buffer and saliva. A possible explanation therefor may be attributed to pH changes that occur during evaporation and drying of microdroplets^11^. The absence of protecting components such as salt crystals or saliva proteins may explain the low survival rate.

This indicates that the physicochemical properties, experienced by the virions in the SM droplets from time of deposition through evaporation until reaching equilibrium (typically within minutes, see Supp. Video 3), affect virus stability and viability. The dramatic increase in salt concentrations, and/or the time of exposure to high salt concentrations and reduced pH, likely cause damage to the virus. This in turn, suggests that the evaporation kinetics of suspended droplets on surfaces may affect the survival of viruses, in a similar manner as that suggested in aerosol droplets^16^. Thus, complete dryness could in fact protect the virions from those high concentrations of dissolved salts and low pH^5,12^.

Another less-understood issue is viral aggregation^56,57^. Spontaneous aggregation of virions, for example as a response to changes in salt concentrations and pH^50,56,58^, may occur in drying microdroplets, and thus may play a role in virus survival. We speculate that with lipid-enveloped viruses suspended in water, aggregation of viruses imposed by hydrophobicity or other mechanisms might play a vital role. If significant viral aggregation occurs, it has implications for interpretation of PFU/mL survival estimates (true for any virus survival study), and for the distribution of virions in drying droplets. Large virion aggregates (> tens of virions) might also affect microdroplets’ drying dynamics due to capillary pinning, as we have shown for bacteria ^28^.

The results of this study provide important insights concerning COVID-19. Although performed with a surrogate for SARS-CoV-2, it indicates, as have other studies, that enveloped viruses like SARS-CoV-2 survive well in evaporated saliva droplets on inanimate surfaces^19,34–39^. Because SARS-CoV-2 transmission and disease severity appear to depend upon viral load^59^, it is likely that in indoor environments where infected individuals stay for long periods, viable viruses persist on fomites for days. Thus, as long as not proved otherwise, indirect transmission through inanimate surfaces—in particular those with prolonged and high contact like mobile phones and touch screens—is not unlikely, and must be considered.

This study provides further evidence that viruses can survive well in evaporated saliva microdroplets for hours after their deposition on surfaces. What makes human saliva a favorable environment for virus survival is not well understood and thus calls for further research. Interdisciplinary research is required to uncover how the physicochemical microenvironment surrounding the virion affects its survival in saliva microdroplets, and may pinpoint specific components of saliva, such as proteins, polysaccharides, salts or surfactants, that are essential for virus survival.

## Methods

### Bacteria and bacteriophage strains

Bacteriophages Phi6 (DSM-21518), PhiX174 (DSM-4497), and MS2 (DSM-13767) were purchased from the German Collection of Microorganisms and Cell Cultures DSMZ. Vacuum-dried phages were revitalized as per DSMZ instructions. Bacteriophage propagation: A two-cycle bacteriophage propagation protocol was used to obtain ~ 10 mL titers of > 10^10^ PFU/mL. Overnight bacterial host cultures were diluted at 1:50 into 1 mL fresh media and grown to OD_600_ 0.3. A single plaque isolate, suspended in 100 µL SM buffer, was used to infect the fresh culture (first propagation cycle). The infected culture was shake incubated at the appropriate temperature for the host strain for 3 h, or until lysis was observed. Meanwhile, the overnight host cultures were diluted again in 9 mL fresh media and grown to OD_600_ 0.3. 1 mL of the first propagation sample was used to infect 9 mL of fresh host cultures (second propagation cycle) that was shake incubated for 3 h or until lysis was observed. The second bacteriophage propagation was centrifuged (4,200_RCF_, 10 min) and filtered through a 0.22 µm filter (Millex GV). Phages were stored at 4 °C and their concentrations were determined by the drop plaque assay. *Pseudomonas syringae* (DSM-21482), a host strain for bacteriophage Phi6, was purchased from DSMZ and cultivated in TSB (Tryptic Soy Broth) at 28 °C. *Escherichia coli* (DSM-13127), a host strain for bacteriophage PhiX174, was purchased from DSMZ and cultivated in LB (Luria–Bertani broth) at 37 °C. *Escherichia coli* (ATCC 15,597) (kindly provided by Adi Stern), a host strain for bacteriophage MS2, was cultivated in LB at 37 °C.

### Viability of bacteriophages in microdroplets deposited on a glass surface

Phage stock solution (10^8^ PFU/mL for Phi6; and MS2 and 10^7^ PFU/mL for PhiX174) were diluted tenfold with: (1) SM Buffer (100 mM NaCl, 8 mM MgSO4 × 7H_2_O, 50 mM Tris–Cl pH 7.5, 0.01% w/v gelatin) (2) filter-sterilized H_2_O (W4502, sigma) and (3) natural human saliva (donated by one of the authors). Fluorescent beads (2 µm, melamine resin labeled by rhodamine B, 94,009 Fluka) were added to the suspension with a 5 × 10^2^ final dilution. We aimed to have an equivalent concentration of beads to those of the Phi6 and MS2 viruses (~ 10^6^/mL). The beads were added for two purposes: (i) to mimic micrometer-sized particulates (e.g., bacteria cells) that are spread on real-world surfaces and have been shown to affect the formation of microscopic surface wetness^28^; and to (ii) help interpret the virion distribution within droplets. Solutions were loaded into 5 mL refillable spray bottles (purchased at a local cosmetics store) and a portion of the load was sprayed on a 12-well glass bottom plate (P12-1.5H-N, Cellvis). Each well was sprayed by pressing the spray nozzle twice (delivering a total volume of ~ 50 µL). Spray was applied to the well through a 50 mL falcon tube from which we chopped 1.5 cm from its conical end. The plates were placed without the cover lid inside a sealed plastic box. As this study’s focus is virus survival in the indoor environment, we chose to work at a temperature of 25 °C and a range of RH (23% to 78%), which spans most indoor environments. A 100 mL saturated salt solution (potassium acetate 268.6 g/100 mL, 23% RH; potassium carbonate 111 g/100 mL, 43% RH; sodium bromide 94.6 g/100 mL, 57% RH; and ammonium chloride 39.5 g/100 mL, 78% RH) was placed in the bottom of each box to maintain relative humidity of 23%, 43%, 57%, and 78%. The boxes were placed in an incubator set at 25 °C for 14 h. Control: spray bottles with the remaining (unsprayed) suspensions were left in incubation for 14 h and sprayed onto the 12-well plates at the end of the incubation period. All plates were imaged at the end of the 14-h incubation period and then suspended with 500 µL of SM buffer for the drop plaque assay.

### Virus plaque assay

Plates containing the bottom agar layer were poured in advance (TSB or LB with 15 g/L agar, 5 mM MgSO_4_, and 5 mM CaCl_2_). On the day of the experiment, overnight bacterial host cultures were diluted at 1:50 into 1 mL fresh media and shake incubated until they reached OD_600_ 0.3. Meanwhile, the top agar (TSB or LB with 7.5 g/L agar, 5 mM MgSO_4_, and 5 mM CaCl_2_) was melted and kept in a 55 °C water bath. The bacterial host culture was combined with the top agar at a ratio of 1:40, and poured on top of the bottom layer. The phages were serially diluted in SM buffer, and after the top agar solidified, either 10 µL, 100 µL, or 1000 µL were pipetted and spread out onto either one quarter, one half, or a full plate (10 × 10 cm petri dish) respectively. Plates were left open until dry, and incubated at the appropriate temperature for the host strain (as described above) overnight.

### Microscopy

12-well plates were mounted on a stage top without warming (room temperature) during image acquisition. Images were collected using an Eclipse Ti-E inverted epi-fluorescence microscope (Nikon) equipped with a Plan Apo 20x/0.75 NA air objective and the Perfect Focus System for maintenance of focus. An LED light source (SOLA SE II, Lumencor) was used for fluorescence excitation. Rhodamine B-marked fluorescent beads were excited through a 560/40 filter, and emission was collected with a T585lpxr dichroic mirror and 630/75 filter (filter cube #49008, Chroma). Images were acquired with a SCMOS camera (ZYLA 4.2PLUS, Andor Technology, Ltd.) controlled with NIS Elements 5.02 software (Nikon Instruments Inc.). 10 × 10 adjacent fields of views (covering a total area of 2.82 × 2.82 mm) were monitored per each well. Multiple stage positions were collected using a motorized encoded scanning stage (SCANplus IM 130 × 85, Märzhäuse).

### Statistical analyses and calculations

#### Statistical analyses

To investigate the relationships between the dependent variable of virus viability (in log10 transformed values) and the independent variable of RH, we fitted statistical models in MATALB using ordinary least-squares regression. We evaluated linear correlations based on R^2^, F-tests, and P-values for statistical significance of the linear models (see Supp. Fig S1), and Correlation Coefficients based on Pearson correlation coefficients r (see Supp. Fig S2).

Statistical significance of the differences in viability reductions between saliva and the other two aqueous media, under equal RH levels, was assessed using Welch t-test (see Supp. Fig S3). Statistical analyses were performed in MATLAB.

#### Estimation of the number of viruses per microdroplet

The initial concentration of viruses was ~ 10^6^ per mL. The original volume of a droplet that left a 100-µm diameter deposition was estimated at ~ 0.01 nL. Assuming there is no significant virus aggregation and that viruses are uniformly distributed, we obtain ~ 10^6^ per mL × 10^–5^ = 10 virions per such microdroplet.

## Supplementary Information

Supplementary Video 1.

Supplementary Video 2.

Supplementary Video 3.

Supplementary Information 1.

## Data Availability

The datasets generated during and/or analyzed during the current study are available in the Figshare repository at https://figshare.com/articles/Virus_survival_in_evaporated_saliva_microdroplets_deposited_on_inanimate_surfaces/12546563.
