# Supplementary material for: Survival of the enveloped bacteriophage Phi6 (a surrogate for SARS-CoV-2) in evaporated saliva microdroplets deposited on glass surfaces
Source: Sci Rep. 2020 Dec 29;10:22419. doi: 10.1038/s41598-020-79625-z (PMC7772334; doi:10.1038/s41598-020-79625-z)
Supplement: Supplementary file 4 — Supplementary Information 1. [file 41598_2020_79625_MOESM4_ESM.pdf]

Survival of the enveloped bacteriophage Phi6 (a surrogate for SARS-CoV-2) in evaporated saliva microdroplets deposited on glass surfaces

Aliza Fedorenko[1], Maor Grinberg[1], Tomer Orevi[1], Nadav Kashtan[1*]

Supplementary Information

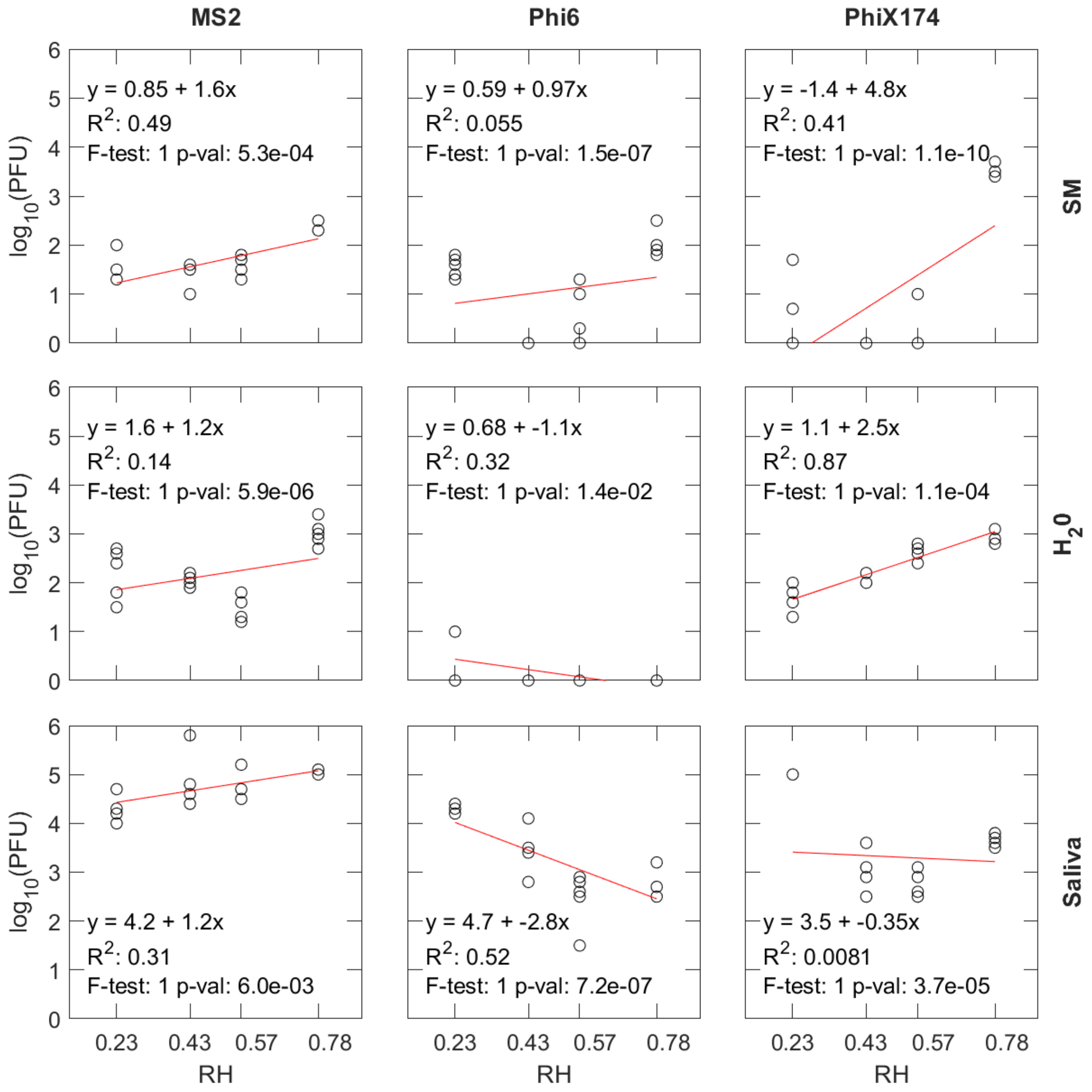

Supp. Fig. S1 Linear regression analysis of reduction in phage viability (Log10 transformed) as a function of RH. Circles mark experimental values, regression line (red line) is shown along with its linear equation, R^2 values, F-statistics and P-values. Data points are same as presented in Fig. 4. Analysis was performed in MATLAB.

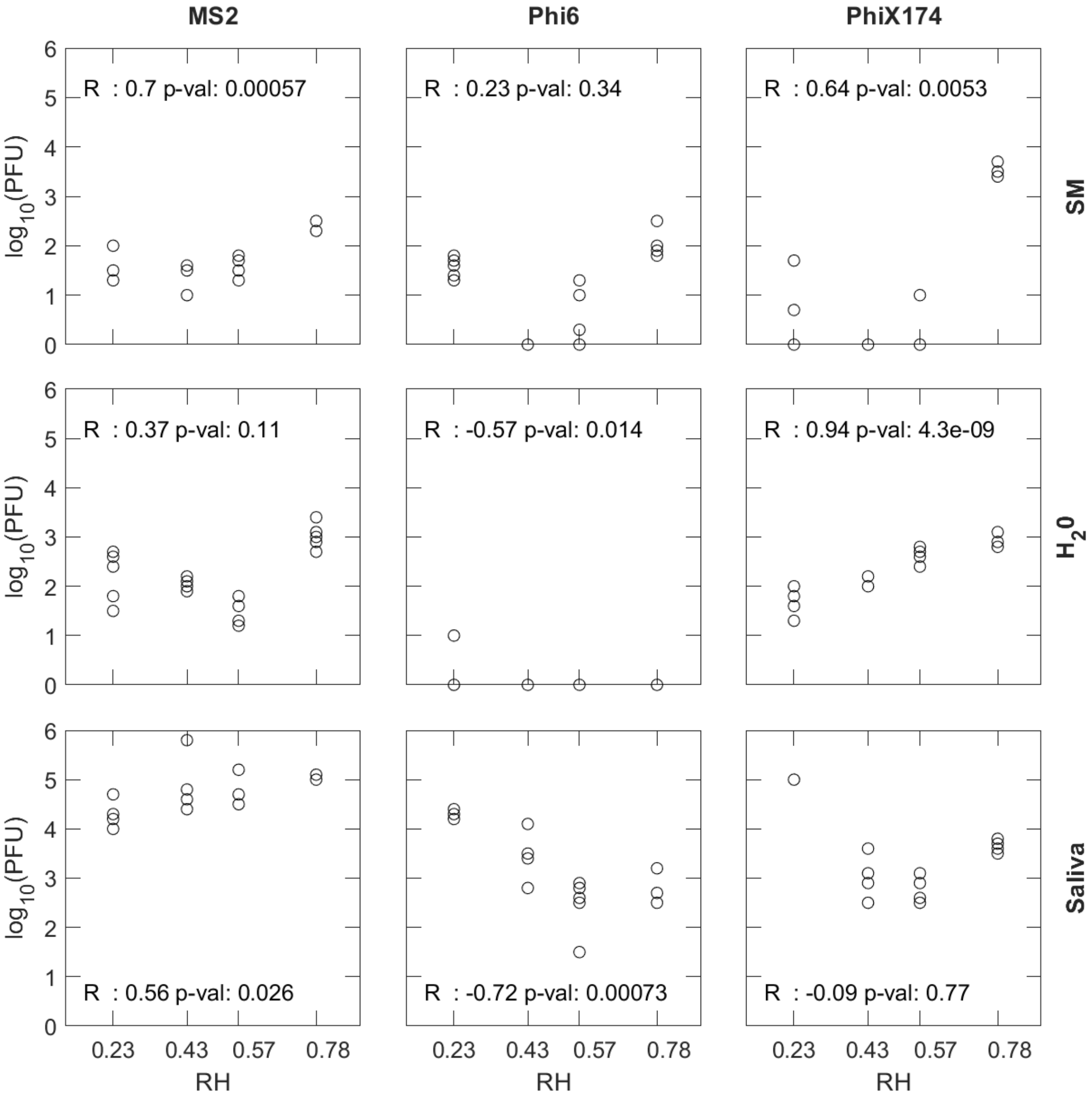

Supp. Fig. S2 Correlation between reduction in phage viability (Log10 transformed) and RH. Circles mark experimental values. R values are Pearson correlation coefficients. Data points are same as presented in Fig. 4. Analysis was performed in MATLAB.

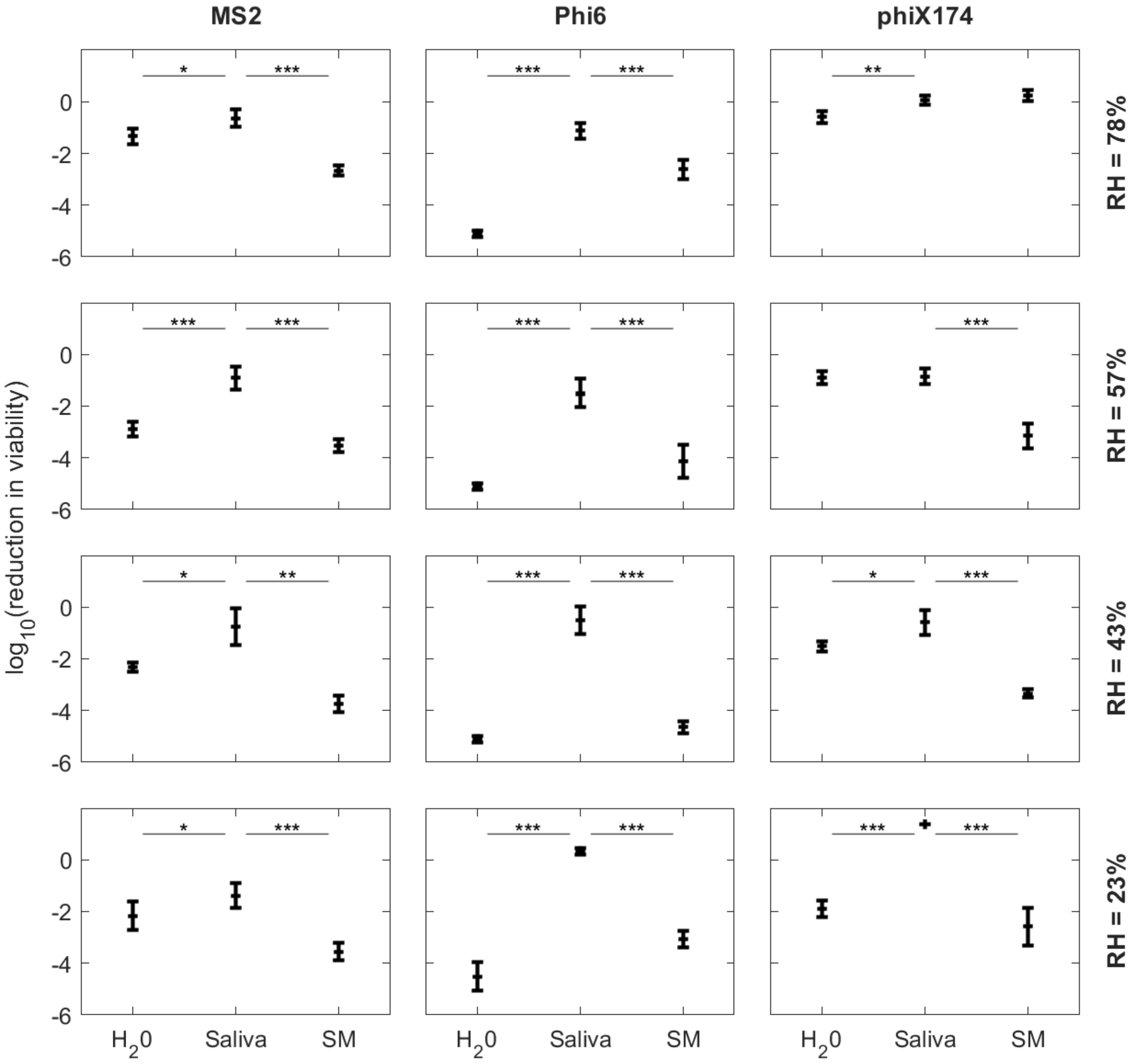

Supp. Fig. S3 virus survival in saliva microdroplets in comparison to SM buffer and water under same RH levels. The significance was assessed by Welch t-test. Significance marked by *, **, or ***, denoting p-values of <0.05, <0.005, or <0.0005, respectively. Data points are same as presented in Fig. 4. Analysis was performed in MATLAB.
